# (*R*)-4-[2-(Methyl­sulfanyl)pyrimidin-4-yl]-1-(tetra­hydro­furan-3-yl)-1*H*-pyrazol-5-amine

**DOI:** 10.1107/S160053680900734X

**Published:** 2009-03-06

**Authors:** Zhengyu Liu, Kevin K.-C. Liu, Jeff Elleraas, Arnold L. Rheingold, Antonio DiPasquale, Alex Yanovsky

**Affiliations:** a Pfizer Global Research and Development, La Jolla Labs, 10614 Science Center Drive, San Diego, CA 92121, USA; b Department of Chemistry and Biochemistry, University of California, San Diego, 9500 Gilman Drive, La Jolla, CA 92093, USA

## Abstract

The title compound, C_12_H_15_N_5_OS, was obtained by reaction of 2-(2-(methyl­thio)pyrimidin-4-yl)-3-oxopropane­nitrile with (tetra­hydro­furan-3-yl)hydrazine dihydro­chloride, and the racemic product was subsequently separated by chiral chromatography (first peak; [α]_D_^20^ = +51.3°). The chiral center at the substituted atom of the tetra­hydro­furanyl group has an *R*-configuration. The pyrimidine and pyrazolyl rings are almost coplanar, their mean planes forming a dihedral angle of 6.4 (1)°. One of the H atoms of the amino group participates in an intra­molecular hydrogen bond with the pyrimidine N atom in position 3. The second H atom is involved in an inter­molecular hydrogen bond, which links the mol­ecules into an infinite chain.

## Related literature

For the structure of a related compound with a methyl-substituted amino group, see: Liu *et al.* (2009 ▶).
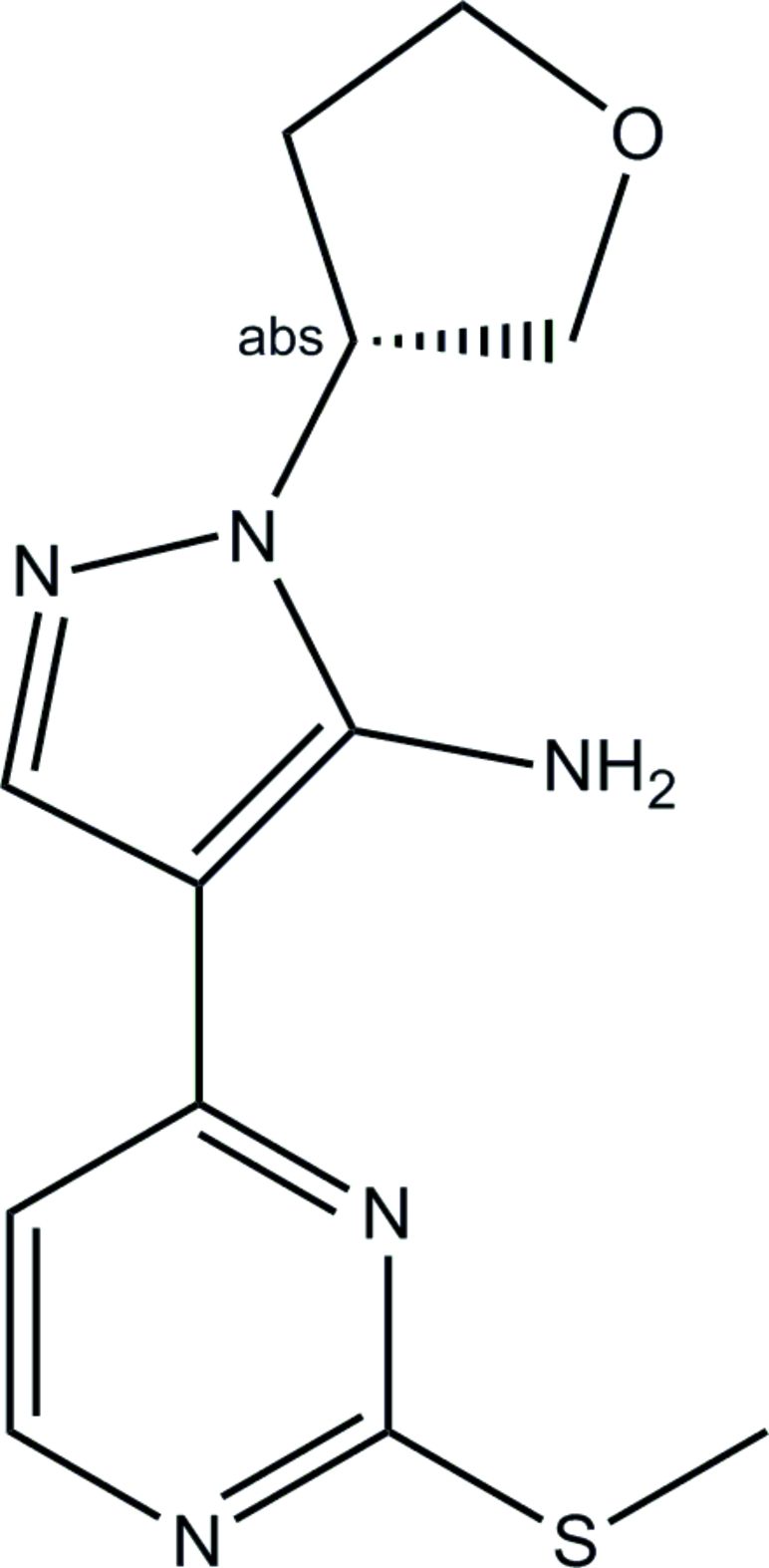


## Experimental

### 

#### Crystal data

C_12_H_15_N_5_OS
*M* *_r_* = 277.35
Orthorhombic, 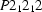

*a* = 15.479 (2) Å
*b* = 7.1217 (10) Å
*c* = 11.7802 (17) Å
*V* = 1298.6 (3) Å^3^
*Z* = 4
Mo *K*α radiation
μ = 0.25 mm^−1^
*T* = 208 K
0.20 × 0.20 × 0.20 mm

#### Data collection

Bruker D8 APEXII CCD area-detector diffractometer
Absorption correction: multi-scan (*SADABS*; Bruker, 2001 ▶) *T*_min_ = 0.844, *T*_max_ = 0.952
6570 measured reflections
3025 independent reflections
2844 reflections with *I* > 2σ(*I*)
*R*_int_ = 0.043

#### Refinement

*R*[*F* ^2^ > 2σ(*F* ^2^)] = 0.041
*wR*(*F* ^2^) = 0.115
*S* = 1.07
3025 reflections
174 parameters
H-atom parameters constrained
Δρ_max_ = 0.24 e Å^−3^
Δρ_min_ = −0.35 e Å^−3^
Absolute structure: Flack (1983 ▶), 1180 Friedel pairs
Flack parameter: −0.05 (8)

### 

Data collection: *APEX2* (Bruker, 2004 ▶); cell refinement: *SAINT* (Bruker, 2004 ▶); data reduction: *SAINT*; program(s) used to solve structure: *SIR2004* (Burla *et al.*, 2005 ▶); program(s) used to refine structure: *SHELXL97* (Sheldrick, 2008 ▶); molecular graphics: *ORTEP-32* (Farrugia, 1997 ▶); software used to prepare material for publication: *WinGX* (Farrugia, 1999 ▶).

## Supplementary Material

Crystal structure: contains datablocks global, I. DOI: 10.1107/S160053680900734X/tk2382sup1.cif

Structure factors: contains datablocks I. DOI: 10.1107/S160053680900734X/tk2382Isup2.hkl

Additional supplementary materials:  crystallographic information; 3D view; checkCIF report

## Figures and Tables

**Table 1:** Hydrogen-bond geometry (Å, °)

*D*—H⋯*A*	*D*—H	H⋯*A*	*D*⋯*A*	*D*—H⋯*A*
N3—H3*A*⋯N4^i^	0.87	2.19	2.9731 (19)	150
N3—H3*B*⋯N5	0.87	2.28	2.8616 (19)	124

Symmetry code: (i) 
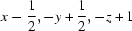
.

**Fig. 1.**
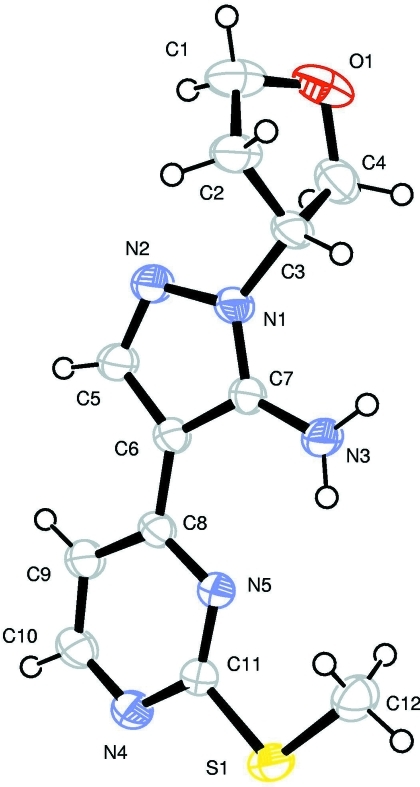
Molecular structure of (I), showing 50% probability displacement ellipsoids and atom numbering scheme. H atoms are drawn as circles with arbitrary small radius.

**Fig. 2.**
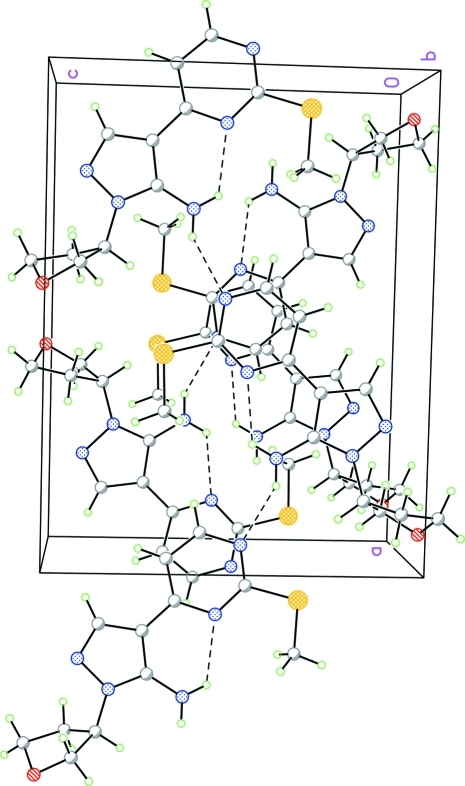
Crystal packing for (I) viewed down the b axis; H-bonds are shown as dashed lines.

## supplementary crystallographic information

### Comment

The title compound was obtained by reaction of 2-(2-(methylthio)pyrimidin-4-yl)-3-oxopropanenitrile with (tetrahydrofuran-3-yl)hydrazine dihydrochloride. The racemic product was then separated with the help of chiral chromatography; the title compound, (I), was collected as the earlier fraction, when eluted with methanol using the Chiralpak column (99% ee; [α]_D_^20^ = +51.3°).

The present X-ray study unambiguously established the *R* configuration of the chiral center at the C3 atom (Fig. 1).

The pyrimidine and pyrazolyl rings lie approximately in one plane; the dihedral angle formed by their mean planes is equal to 6.4 (1)°. The orientation of the tetrahydrofurane ring can be characterized by the dihedral angle 99.6 (1)° formed by the pyrazolyl plane with the C2—C3—C4 plane.

The molecular geometry of (I) is similar to that of related compound with a methyl substituent at the amino group (Liu *et al.*, 2009). However, the crystal packing is substantially different as (I) has one additional H atom capable of H-bond formation. Indeed, while the H3A atom forms an intramolecular H-bond with the N5 atom of the pyrimidine ring similar to that observed in methyl-substituted structure, the H3B atom is involved in intermolecular H-bonding, which links molecules into infinite chains running along the *a* axis (Fig. 2; Table 2).

### Experimental

To a suspension of 2-(2-(methylthio)pyrimidin-4-yl)-3-oxopropanenitrile (13.5 g, 70.0 mmol) in AcOH (100 ml) was added (tetrahydrofuran-3-yl)hydrazine dihydrochloride (12.3 g,70.0 mmol), and the resulting orange mixture was heated at 80°C under nitrogen for 3 h. Acetic acid was removed and the orange solid residue was partitioned between aqueous Na_2_CO_3_ (200 ml) and EtOAc (400 ml). The mixture was refluxed for 30 min. The separated organic layer was washed with brine, dried over sodium sulfate and concentrated to give the crude product as a brown gum (16.82 g, 87%). The brown gum (8.32 g) was purified by flash chromatography using 30–70% EtOAc in hexane to afford a yellow solid (5.96 g).

The part of the product thus obtained (4.85 g) was subjected to chiral chromatography on Chiralpak AS—H 21.2 *x* 250 mm column with 35% MeOH in CO_2_ at 140 bar as eluent (flow = 55 ml/min; UV detection at 260 nm). Two fractions corresponding to each of the enantiomers (Peak1 and Peak2) were collected and evaporated to dryness; the specific rotation [α]_D_^20^ was measured in CH_2_Cl_2_ solution and yielded the values of +51.3° and -52.1°, respectively. The enantiomer collected as Peak 1 was recrystallized from EtOAc/hexane to yield colorless single crystals.

### Refinement

All H atoms were placed in geometrically calculated positions (C—H 0.94 Å, 0.97 Å, 0.98 Å, and 0.99 Å for aromatic-, methyl-, methylene- and methine-H atoms, respectively; N—H 0.87 Å) and included in the refinement in the riding model approximation. The *U*_iso_(H) values were set to 1.2*U*_eq_ of the carrying atom except for 1.5*U*_eq_ for methyl-H atoms.

### Crystal data

**Table:**

C_12_H_15_N_5_OS	*F*(000) = 584
*M**_r_* = 277.35	*D*_x_ = 1.419 Mg m^−^^3^
Orthorhombic, *P*2_1_2_1_2	Mo *K*α radiation, λ = 0.71073 Å
Hall symbol: P 2 2ab	Cell parameters from 5004 reflections
*a* = 15.479 (2) Å	θ = 2.6–28.1°
*b* = 7.1217 (10) Å	µ = 0.25 mm^−^^1^
*c* = 11.7802 (17) Å	*T* = 208 K
*V* = 1298.6 (3) Å^3^	Block, yellow
*Z* = 4	0.20 × 0.20 × 0.20 mm

### Data collection

**Table:**

Bruker D8 APEXII CCD area-detector diffractometer	3025 independent reflections
Radiation source: fine-focus sealed tube	2844 reflections with *I* > 2σ(*I*)
graphite	*R*_int_ = 0.043
phi and ω scans	θ_max_ = 28.1°, θ_min_ = 1.7°
Absorption correction: multi-scan (*SADABS*; Bruker, 2001)	*h* = −20→19
*T*_min_ = 0.844, *T*_max_ = 0.952	*k* = −4→9
6570 measured reflections	*l* = −15→13

### Refinement

**Table:**

Refinement on *F*^2^	Hydrogen site location: inferred from neighbouring sites
Least-squares matrix: full	H-atom parameters constrained
*R*[*F*^2^ > 2σ(*F*^2^)] = 0.041	*w* = 1/[σ^2^(*F*_o_^2^) + (0.0703*P*)^2^ + 0.0815*P*] where *P* = (*F*_o_^2^ + 2*F*_c_^2^)/3
*wR*(*F*^2^) = 0.115	(Δ/σ)_max_ = 0.001
*S* = 1.07	Δρ_max_ = 0.24 e Å^−^^3^
3025 reflections	Δρ_min_ = −0.35 e Å^−^^3^
174 parameters	Extinction correction: *SHELXL* (Sheldrick, 2008), Fc^*^=kFc[1+0.001xFc^2^λ^3^/sin(2θ)]^-1/4^
0 restraints	Extinction coefficient: 0.058 (5)
Primary atom site location: structure-invariant direct methods	Absolute structure: Flack (1983), 1180 Friedel pairs
Secondary atom site location: difference Fourier map	Flack parameter: −0.05 (8)

### Special details

**Table:**

Geometry. All e.s.d.'s (except the e.s.d. in the dihedral angle between two l.s. planes) are estimated using the full covariance matrix. The cell e.s.d.'s are taken into account individually in the estimation of e.s.d.'s in distances, angles and torsion angles; correlations between e.s.d.'s in cell parameters are only used when they are defined by crystal symmetry. An approximate (isotropic) treatment of cell e.s.d.'s is used for estimating e.s.d.'s involving l.s. planes.
Refinement. Refinement of *F*^2^ against ALL reflections. The weighted *R*-factor *wR* and goodness of fit *S* are based on *F*^2^, conventional *R*-factors *R* are based on *F*, with *F* set to zero for negative *F*^2^. The threshold expression of *F*^2^ > σ(*F*^2^) is used only for calculating *R*-factors(gt) *etc*. and is not relevant to the choice of reflections for refinement. *R*-factors based on *F*^2^ are statistically about twice as large as those based on *F*, and *R*- factors based on ALL data will be even larger.

### Fractional atomic coordinates and isotropic or equivalent isotropic displacement parameters (Å^2^)

**Table:**

	*x*	*y*	*z*	*U*_iso_*/*U*_eq_	
C1	0.61092 (15)	0.3862 (4)	1.04598 (18)	0.0592 (6)	
H1A	0.6660	0.3550	1.0825	0.071*	
H1B	0.5758	0.4600	1.0989	0.071*	
C2	0.62567 (13)	0.4931 (3)	0.93597 (14)	0.0471 (4)	
H2A	0.6760	0.5761	0.9418	0.057*	
H2B	0.5748	0.5677	0.9154	0.057*	
C3	0.64143 (10)	0.3354 (3)	0.85008 (15)	0.0414 (4)	
H3	0.6054	0.3562	0.7818	0.050*	
C4	0.61086 (12)	0.1571 (3)	0.91240 (18)	0.0514 (5)	
H4A	0.5721	0.0835	0.8639	0.062*	
H4B	0.6603	0.0785	0.9334	0.062*	
C5	0.86860 (11)	0.3275 (3)	0.84766 (15)	0.0456 (4)	
H5	0.9228	0.3341	0.8835	0.055*	
C6	0.85782 (9)	0.3006 (3)	0.72974 (14)	0.0355 (4)	
C7	0.76781 (9)	0.3006 (2)	0.71461 (13)	0.0341 (3)	
C8	0.92276 (9)	0.2767 (2)	0.64376 (13)	0.0341 (3)	
C9	1.01122 (10)	0.2623 (3)	0.66996 (14)	0.0427 (4)	
H9	1.0308	0.2671	0.7454	0.051*	
C10	1.06733 (10)	0.2412 (3)	0.58192 (16)	0.0466 (4)	
H10	1.1264	0.2289	0.5987	0.056*	
C11	0.95801 (10)	0.2531 (3)	0.45560 (14)	0.0371 (3)	
C12	0.81445 (13)	0.2818 (3)	0.31648 (16)	0.0526 (5)	
H12A	0.7888	0.1785	0.3583	0.079*	
H12B	0.7917	0.2833	0.2398	0.079*	
H12C	0.8006	0.3993	0.3539	0.079*	
N1	0.73257 (8)	0.3241 (2)	0.81741 (12)	0.0393 (3)	
N2	0.79452 (9)	0.3423 (3)	0.90197 (12)	0.0488 (4)	
N3	0.72268 (9)	0.2815 (3)	0.61696 (12)	0.0493 (4)	
H3A	0.6665	0.2841	0.6182	0.059*	
H3B	0.7498	0.2667	0.5529	0.059*	
N4	1.04322 (9)	0.2367 (3)	0.47231 (13)	0.0453 (4)	
N5	0.89635 (8)	0.2704 (2)	0.53396 (11)	0.0341 (3)	
O1	0.56646 (9)	0.2206 (3)	1.01145 (14)	0.0630 (5)	
S1	0.92940 (3)	0.25258 (9)	0.31179 (4)	0.05191 (18)	

### Atomic displacement parameters (Å^2^)

**Table:**

	*U*^11^	*U*^22^	*U*^33^	*U*^12^	*U*^13^	*U*^23^
C1	0.0580 (12)	0.0835 (15)	0.0362 (10)	0.0152 (11)	0.0142 (8)	0.0072 (10)
C2	0.0412 (9)	0.0661 (12)	0.0340 (8)	0.0096 (8)	0.0063 (7)	0.0034 (8)
C3	0.0248 (7)	0.0669 (11)	0.0325 (8)	0.0036 (7)	0.0002 (6)	0.0017 (8)
C4	0.0343 (8)	0.0675 (12)	0.0523 (11)	−0.0037 (8)	0.0026 (7)	0.0040 (10)
C5	0.0288 (7)	0.0790 (13)	0.0290 (7)	−0.0006 (8)	−0.0025 (6)	−0.0024 (8)
C6	0.0258 (7)	0.0512 (9)	0.0293 (7)	−0.0007 (6)	−0.0009 (5)	0.0000 (7)
C7	0.0269 (7)	0.0473 (8)	0.0282 (7)	0.0005 (6)	−0.0020 (5)	−0.0004 (6)
C8	0.0262 (6)	0.0443 (8)	0.0320 (7)	−0.0002 (6)	0.0010 (5)	0.0008 (6)
C9	0.0277 (7)	0.0655 (11)	0.0350 (7)	0.0007 (7)	−0.0029 (6)	0.0006 (9)
C10	0.0259 (7)	0.0684 (11)	0.0456 (9)	0.0031 (9)	0.0023 (6)	−0.0035 (9)
C11	0.0315 (7)	0.0471 (8)	0.0327 (7)	−0.0020 (7)	0.0027 (5)	−0.0045 (8)
C12	0.0438 (9)	0.0754 (13)	0.0385 (9)	0.0067 (9)	−0.0061 (7)	−0.0041 (10)
N1	0.0254 (6)	0.0642 (9)	0.0282 (6)	0.0003 (6)	−0.0011 (5)	−0.0007 (6)
N2	0.0290 (6)	0.0884 (12)	0.0290 (7)	−0.0003 (7)	−0.0043 (5)	−0.0034 (8)
N3	0.0284 (6)	0.0917 (13)	0.0278 (6)	0.0010 (7)	−0.0043 (5)	−0.0065 (8)
N4	0.0293 (6)	0.0657 (9)	0.0408 (7)	−0.0002 (7)	0.0045 (5)	−0.0076 (9)
N5	0.0287 (6)	0.0435 (7)	0.0300 (6)	−0.0016 (5)	0.0020 (4)	−0.0026 (6)
O1	0.0460 (8)	0.0826 (11)	0.0604 (9)	0.0041 (8)	0.0233 (6)	0.0185 (8)
S1	0.0394 (2)	0.0852 (4)	0.0311 (2)	−0.0016 (2)	0.00439 (15)	−0.0075 (2)

### Geometric parameters (Å, °)

**Table:**

C1—O1	1.425 (3)	C7—N1	1.339 (2)
C1—C2	1.520 (3)	C7—N3	1.353 (2)
C1—H1A	0.9800	C8—N5	1.357 (2)
C1—H1B	0.9800	C8—C9	1.407 (2)
C2—C3	1.531 (3)	C9—C10	1.361 (2)
C2—H2A	0.9800	C9—H9	0.9400
C2—H2B	0.9800	C10—N4	1.344 (2)
C3—N1	1.4644 (18)	C10—H10	0.9400
C3—C4	1.541 (3)	C11—N5	1.3334 (19)
C3—H3	0.9900	C11—N4	1.339 (2)
C4—O1	1.428 (3)	C11—S1	1.7510 (17)
C4—H4A	0.9800	C12—S1	1.792 (2)
C4—H4B	0.9800	C12—H12A	0.9700
C5—N2	1.317 (2)	C12—H12B	0.9700
C5—C6	1.412 (2)	C12—H12C	0.9700
C5—H5	0.9400	N1—N2	1.3888 (18)
C6—C7	1.4046 (19)	N3—H3A	0.8700
C6—C8	1.437 (2)	N3—H3B	0.8700
			
O1—C1—C2	104.11 (19)	N1—C7—C6	106.83 (13)
O1—C1—H1A	110.9	N3—C7—C6	128.32 (15)
C2—C1—H1A	110.9	N5—C8—C9	119.98 (14)
O1—C1—H1B	110.9	N5—C8—C6	117.70 (13)
C2—C1—H1B	110.9	C9—C8—C6	122.32 (14)
H1A—C1—H1B	109.0	C10—C9—C8	117.50 (15)
C1—C2—C3	102.70 (18)	C10—C9—H9	121.2
C1—C2—H2A	111.2	C8—C9—H9	121.2
C3—C2—H2A	111.2	N4—C10—C9	123.87 (14)
C1—C2—H2B	111.2	N4—C10—H10	118.1
C3—C2—H2B	111.2	C9—C10—H10	118.1
H2A—C2—H2B	109.1	N5—C11—N4	127.69 (15)
N1—C3—C2	111.55 (15)	N5—C11—S1	119.28 (12)
N1—C3—C4	112.07 (16)	N4—C11—S1	113.03 (12)
C2—C3—C4	103.93 (14)	S1—C12—H12A	109.5
N1—C3—H3	109.7	S1—C12—H12B	109.5
C2—C3—H3	109.7	H12A—C12—H12B	109.5
C4—C3—H3	109.7	S1—C12—H12C	109.5
O1—C4—C3	106.03 (18)	H12A—C12—H12C	109.5
O1—C4—H4A	110.5	H12B—C12—H12C	109.5
C3—C4—H4A	110.5	C7—N1—N2	112.28 (13)
O1—C4—H4B	110.5	C7—N1—C3	129.58 (14)
C3—C4—H4B	110.5	N2—N1—C3	118.13 (13)
H4A—C4—H4B	108.7	C5—N2—N1	104.20 (14)
N2—C5—C6	112.69 (15)	C7—N3—H3A	120.0
N2—C5—H5	123.7	C7—N3—H3B	120.0
C6—C5—H5	123.7	H3A—N3—H3B	120.0
C7—C6—C5	104.00 (14)	C11—N4—C10	114.37 (14)
C7—C6—C8	127.19 (14)	C11—N5—C8	116.57 (13)
C5—C6—C8	128.81 (14)	C1—O1—C4	105.25 (15)
N1—C7—N3	124.85 (14)	C11—S1—C12	102.76 (8)

### Hydrogen-bond geometry (Å, °)

**Table:**

*D*—H···*A*	*D*—H	H···*A*	*D*···*A*	*D*—H···*A*
N3—H3A···N4^i^	0.87	2.19	2.9731 (19)	150
N3—H3B···N5	0.87	2.28	2.8616 (19)	124

Symmetry codes: (i) *x*−1/2, −*y*+1/2, −*z*+1.
